# TLR2 stimulation impairs anti-inflammatory activity of M2-like macrophages, generating a chimeric M1/M2 phenotype

**DOI:** 10.1186/s13075-017-1447-1

**Published:** 2017-11-02

**Authors:** Lilian Quero, Edveena Hanser, Tobias Manigold, André N. Tiaden, Diego Kyburz

**Affiliations:** 1 Experimental Rheumatology, University Hospital Basel, Basel, Switzerland; 2 Department of Biomedicine, University of Basel, Petersplatz 10, 4051 Basel, Switzerland

**Keywords:** TLRs, Monocytes, Macrophages, Cytokines, Gene expression, CD markers, NF-κB, MAPKs, IRF3/7, Rheumatoid arthritis

## Abstract

**Background:**

Toll-like receptors (TLRs) and macrophages play an important role in rheumatoid arthritis (RA). Currently, it is not clear whether inflammatory M1 or anti-inflammatory M2 predominate among the resident macrophages in the synovium. In the present study, we set out to investigate the impact of TLR stimulation on monocyte-derived M1 and M2 macrophage function and phenotype by mimicking the exposure to abundant TLR agonists as occurs in the context of RA. The response of macrophage subsets to TLR2 and TLR4 activation was evaluated on cluster of differentiation (CD) marker profile; cytokine secretion; gene expression; and NF-κB, interferon regulatory factors 3 and 7 (IRF3/7), and mitogen-activated protein kinase (MAPK) activation.

**Methods:**

Human monocytes were isolated from peripheral blood of healthy individuals and patients with RA and differentiated into M1-like and M2-like macrophages by granulocyte-macrophage colony-stimulating factor (GM-CSF) and macrophage colony-stimulating factor (M-CSF), respectively. Cells were either (1) stimulated with TLR ligands Pam3 or lipopolysaccharide (LPS) or (2) classically activated via interferon (IFN)-γ/LPS. Cytokine production was measured by enzyme-linked immunosorbent assay, and gene expression was measured by qPCR. Cells were stained for CD markers and analyzed by fluorescence-activated cell sorting. NF-κB, IRF3/7, and MAPKs were detected by Western blotting.

**Results:**

Monocyte-derived macrophages of healthy donors (HD) or patients with RA displayed comparable subset-specific phenotypes upon exposure to TLR agonists. CD14 and CD163 marker expression on M2 macrophages did not change upon TLR2 and TLR4 engagement. By contrast, M2 gene markers *HMOX1*, *FOLR2*, and *SLC40A1* were decreased. Importantly, M2 macrophages derived from HD or patients with RA showed both a decreased ratio of interleukin (IL)-10/IL-6 and IL-10/IL-8 upon stimulation with TLR2 ligand Pam3 compared with TLR4 ligand LPS. Gene expression of TLR2 was increased, whereas TLR4 expression was decreased, by TLR ligand stimulation. MAPKs p38, extracellular signal-regulated kinase 1/2, and c-Jun N-terminal kinase were activated more strongly in M2 than in M1 macrophages by Pam3 or LPS.

**Conclusions:**

We show that the anti-inflammatory activity of M2 macrophages is reduced in the presence of abundant TLR2 ligands without significant changes in cell surface markers. Thus, the classical M1/M2 paradigm based on cellular markers does not apply to macrophage functions in inflammatory conditions such as RA.

**Electronic supplementary material:**

The online version of this article (doi:10.1186/s13075-017-1447-1) contains supplementary material, which is available to authorized users.

## Background

Rheumatoid arthritis (RA) is characterized by synovial inflammation [1] as well as the production of autoantibodies such as rheumatoid factor and antibodies against self-proteins (anticitrullinated peptide antibodies [ACPA]) that underwent citrullination, a posttranslational modification generated by the peptidylarginine deiminase [2]. The synovial tissue in inflamed joints undergoes hyperplasia and forms the so-called pannus [3]. It is well described that activated synovial fibroblasts within the pannus strongly contribute to inflammation and tissue degeneration [4–6]. Furthermore, there is an infiltration of immune cells, such as B cells, T cells, dendritic cells, and macrophages, into the synovium. These activated inflammatory cells produce cytokines, chemokines, matrix metalloproteinases (MMPs), and osteoclast-promoting factors [7–9], resulting in perpetuation of the inflammation, cartilage damage, and bone destruction that are characteristic of RA.

Toll-like receptors (TLRs) have been shown to contribute to the inflammatory response in RA [10, 11]. A few TLRs (TLR2, TLR3, TLR4) have been found to be upregulated in synovial tissue in RA [12] but not in osteoarthritis [13, 14] or in synovial versus peripheral monocytes from patients with RA [15]. Upregulation of TLR2 and TLR4 has been demonstrated in synovial macrophages from patients with RA but not in monocyte-derived macrophages from healthy donors (HD) [16]. Synovial macrophages from patients with RA also showed increased tumor necrosis factor (TNF)-α and interleukin (IL)-8 expression, mediated through TLR2 and TLR4, compared with macrophages from patients with other forms of inflammatory arthritis.

Granulocyte-macrophage colony-stimulating factor (GM-CSF) and macrophage colony-stimulating factor (M-CSF) expression are known to be upregulated at sites of inflammation and autoimmunity [17], and both are increased in synovial fluid (SF) of patients with RA. Specifically, elevated levels of TNF-α and IL-1β in patients with RA can promote the production of GM-CSF as well as M-CSF by synovial fibroblasts and chondrocytes [17–19]. Several studies and reports have demonstrated that GM-CSF-, interferon (IFN)-γ-, lipopolysaccharide (LPS)-, and TNF-α-differentiated monocytes display inflammatory M1 properties, whereas M-CSF, immunoglobulin G (IgG), IL-10, IL-4, and IL-13 lead to anti-inflammatory M2 macrophages [20, 21]. Furthermore, cluster of differentiation 14 (CD14) and CD163 [20] as well as gene markers such as heme oxygenase 1 (*HMOX1*), folate receptor β (*FOLR2*), or solute carrier family 40 member 1 (*SLC40A1*) characterize anti-inflammatory M2 macrophages [22, 23]. Resident macrophages in RA were shown to exhibit a more M1-like proinflammatory activity; however, they also express M2 markers such as CD163 or *HMOX1* [24, 25]. Hence, it remains unclear whether classical M1 or M2 or an as yet undefined macrophage population predominates numerically and functionally in RA [19, 26, 27]. The process of M1 and M2 polarization displays a high grade of plasticity [28], and the phenotype and activation state of polarized macrophages can be altered in a special local microenvironment or can even be reversed under pathophysiological conditions. In our study, we aimed at assessing the functional plasticity of conventional macrophage subsets under inflammatory conditions usually present in RA, such as abundant TLR ligands in synovia as a result of increased tissue damage [1]. We therefore investigated “naive” monocytes from peripheral blood of healthy individuals or patients with RA and differentiated them into M1-like and M2-like macrophages in vitro by using GM-CSF or M-CSF, respectively. These polarized macrophage populations were then challenged with different TLR ligands (Pam3, LPS) and compared with classical cytokine activation via IFN-γ/LPS. To evaluate the functional and phenotypical reaction of the generated M1 and M2 subsets on TLR stimulation, we assessed cytokine release, expression of characteristic gene markers, and alteration in cell surface markers.

We report that TLR2 engagement impairs the anti-inflammatory activity of M2-like macrophages derived from healthy or RA monocytes without changing the expression profile of the conventional M2 cell surface markers CD14 and CD163, but altering the expression of M2-specific gene markers *HMOX1*, *FOLR2*, and *SLC40A1* toward an M1-specific profile. Thus, our study implies the emergence of a “chimeric” M2 subset that exerts decreased anti-inflammatory functions and possibly even constitutes a factor that promotes the inflammatory conditions in a disease setting such as RA.

## Methods

### Isolation, in vitro differentiation, and stimulation of monocytes and monocyte-derived macrophages

Monocytes were isolated from peripheral blood donated from healthy individuals (blood supply center, SRK beider Basel, Basel, Switzerland) or patients with RA (Department of Rheumatology, University Hospital Basel, Basel, Switzerland). RA was determined as defined by the 2010 American College of Rheumatology/European League Against Rheumatism classification criteria. All blood donors gave informed consent to participate in the study. The studies were approved by the regional ethics review board. Monocytes were isolated from peripheral blood mononuclear cells by CD14 microbead separation (Miltenyi Biotec GmbH, Bergisch Gladbach, Germany) and differentiated into M1-like and M2-like macrophages by culturing them in standard medium [RPMI 1640, 10% FCS, 1% glutamine, 1% antibiotics, 1% 4-(2-hydroxyethyl)-1-piperazineethanesulfonic acid (HEPES)] in the presence of 50 ng/ml GM-CSF or M-CSF (PeproTech, Hamburg, Germany), respectively, for 8–10 days. Freshly prepared GM-CSF and M-CSF medium was added every 2–3 days. For M0, CD14^+^ separated cells were either directly processed for surface marker staining or kept in standard medium for 1–3 days for subsequent TLR stimulation experiments. Stimulation of cells was performed for 24 h with 300 ng/ml Pam3CysSerLys4 (Pam3), 100 ng/ml LPS, or 10 μg/ml polyinosinic-polycytidylic acid [poly(I:C)] (all from InvivoGen, San Diego, CA, USA). We used IFN-γ/LPS (20 ng/ml and 100 ng/ml, respectively; PeproTech) as a macrophage activation control.

### Fluorescence-activated cell sorting analysis

After stimulation, cells were washed once with cold filtered PBS/0.5% bovine serum albumin (fluorescence-activated cell sorting [FACS] buffer) and stained with fluorescently labeled antibodies CD14-allophycocyanin-cyanine 7 (APC-Cy7), CD163-fluorescein isothiocyanate (FITC), CD206-BV421, CD86-phycoerythrin (PE), and CD80-FITC (all from BD Biosciences, Allschwil, Switzerland) for 30 minutes on ice in the dark. Cells were then washed three times with FACS buffer and fixed with 1% formaldehyde in FACS buffer. Cells were analyzed by FACS (BD Fortessa; BD Biosciences, San Jose, CA, USA) using FlowJo software (FlowJo, Ashland, OR, USA) for analysis. Results are presented as either the percentage of positive stained cells among the total cell population or as mean fluorescence intensity (MFI), calculated as ΔMFI = MFI_specific surface marker_ − MFI_corresponding unstained control_ and normalized to the basal MFI of unstained control cells.

### Gene expression

Monocytes (M0) or M1- and M2-differentiated macrophages were cultured in 24- or 48-well plates with 270,000 cells/well or 150,000 cells/well, respectively, and stimulated as described above. RNA was isolated using the miRNeasy Micro kit (Qiagen, Hilden, Germany), and 1 μg total RNA was reverse-transcribed with the RealMasterScript SuperMix Kit (5Prime GmbH, Hilden, Germany). Gene expression of *TLR2*, *TLR3*, *TLR4*, *HMOX1*, *FOLR2*, and *SLC40A1* was measured by qRT-PCR using TaqMan StepOnePlus (Applied Biosystems/Thermo Fisher Scientific, Foster City, CA, USA). Values were normalized to ubiquitin C (*UBC*) or TATA-box binding protein (*TBP*) messenger RNA (mRNA) levels and are presented as 2^−ΔCT^.

### Enzyme-linked immunosorbent assay

Cells were cultured in either 48- or 96-well plates with either 150,000 cells/well or 50,000 cells/well, respectively. After stimulation, supernatants were collected and cytokine, chemokine, and MMP3 release was measured by enzyme-linked immunosorbent assay (ELISA) (IL-6, IL-8, IL-1β, and TNF-α ELISAs, eBioscience, San Diego, CA, USA; MMP3 ELISA, R&D Systems, Minneapolis, MN, USA). Values are presented either as concentration in picograms per milliliter or as a ratio.

### Western blot analysis

Monocytes (4 × 10^6^) were cultured in 60 × 15-mm dishes under M1- or M2-like differentiation conditions as described above and stimulated either (1) for 1 h to assess nuclear shuttling of NF-κB and interferon regulatory factors 3 and 7 (IRF3/7) or (2) for 30 minutes for mitogen-activated protein kinase (MAPK) phosphorylation detection. Whole-cell protein extracts were isolated by adding 300 μl of lysis buffer containing 50 mM HEPES (pH 7.5), 450 mM NaCl, 15% glycerol, 2 mM ethylenediaminetetraacetic acid, 1 mM phenylmethylsulfonyl fluoride (PMSF), and freshly added protease/phosphatase inhibitor cocktail (Cell Signaling Technology, Danvers, MA, USA). Protein was harvested by incubation on a roller shaker for 10 minutes and subsequent centrifugation for 30 minutes at 14,000 rpm. For nuclear extraction, cells were washed with cold PBS and lysed with lysis buffer containing 10 mM HEPES (pH 7.9), 1.5 mM MgCl_2_, 10 mM KCl, 1 mM PMSF, 5 mM dithiothreitol (DTT), and 0.1% IGEPAL (Sigma-Aldrich, Buchs, Switzerland) with freshly added protease/phosphatase inhibitor cocktail for 5 minutes on ice. Nuclear pellets were harvested after centrifugation at 10,000 rpm for 5 minutes at 4 °C and lysed for 20 minutes in buffer containing 20 mM HEPES (pH 7.9), 1.5 mM MgCl_2_, 420 mM NaCl, 25% glycerol, 1 mM PMSF, and 5 mM DTT with freshly added protease/phosphatase inhibitor cocktail. Protein concentrations were determined using Coomassie Plus Protein Assay reagent (Thermo Fisher Scientific, Rockford, IL, USA). Equal amounts of protein were loaded onto 12% sodium dodecyl sulfate-PAGE gels and transferred onto PVDF membranes (Bio-Rad Laboratories AG, Cressier, Switzerland). Membranes were incubated over night at 4 °C with either rabbit anti-p38, rabbit anti-phospho-p38 (Thr180/Tyr182), mouse anti-extracellular signal-regulated kinase 1/2 (anti-ERK1/2), rabbit anti-phospho-ERK1/2 (Thr202/Tyr204), rabbit anti-stress-activated protein kinase (SAPK)/c-Jun N-terminal kinase (JNK), rabbit anti-phospho-SAPK/JNK (Thr183/Tyr185), mouse anti-NF-κB p65, rabbit anti-IRF3, rabbit anti-IRF7, and mouse anti-histone H3 (all 1:1000; Cell Signaling Technology). After three washes with Tris-buffered saline with Tween 20 (TBS-T) (0.05% Tween; ROTH AG, Arlesheim, Switzerland) for 10 minutes, membranes were incubated for 1 h with secondary goat anti-rabbit IgG IR800 antibody or goat anti-mouse IgG IR700 antibody (all 1:10,000; Azure Biosystems, Dublin, CA, USA) and washed again three times for 10 minutes with TBS-T and additionally for 5 minutes with TBS before analysis. Fluorescence was detected using an Odyssey CLx imaging system (LI-COR Biosciences GmbH, Bad Homburg vor der Höhe, Germany).

### Statistical analysis

All statistical analyses were carried out using Prism 7 software (GraphPad Software, La Jolla, CA, USA). Data distribution was first assessed for normality using the Kolmogorov-Smirnov test. Parametric analysis of normally distributed data was performed by ordinary one-way analysis of variance (ANOVA) using Dunnett’s multiple comparisons test. Nonparametric data were analyzed using the Kruskal-Wallis test with Dunn’s multiple comparisons test. Multiple-group analysis was carried out by ordinary two-way ANOVA using the Holm-Sidak multiple comparisons test. A *p* value < 0.05 was considered statistically significant. All data are presented as mean ± SD.

## Results

### Expression profile of surface markers on M0, M1-, and M2-polarized macrophages following TLR ligand exposure and activation

**Fig. 1:**
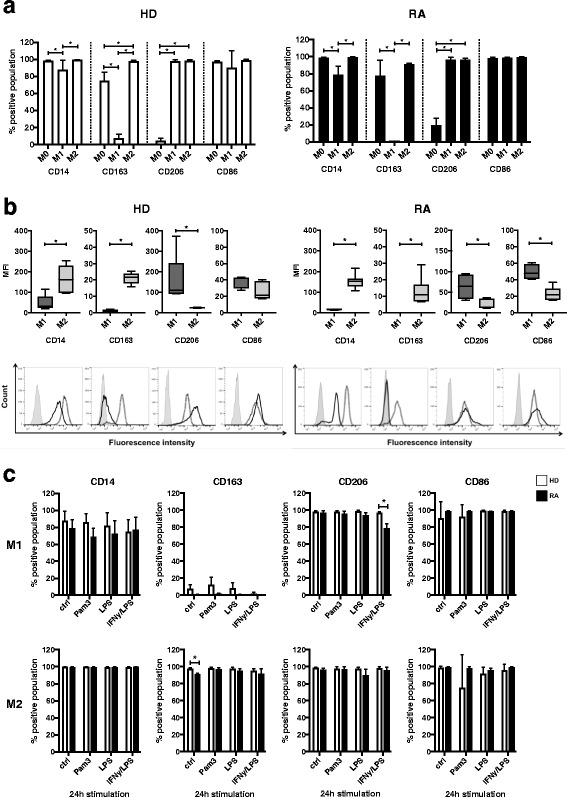
Characterization of surface markers on M0, M1-, and M2-polarized macrophages following Toll-like receptor (TLR) ligand exposure and activation. For phenotypical analysis, M0 (ex vivo monocytes), M1-like (GM-CSF-differentiated), and M2-like (M-CSF-differentiated) macrophages derived from peripheral blood of healthy donors (HD) or patients with rheumatoid arthritis (RA) were stained for fluorescence-activated cell sorting analysis with fluorescently labeled antibodies CD14-allophycocyanin-cyanine 7 (APC-Cy7), CD163-fluorescein isothiocyanate (FITC), CD206-BV421, CD86-phycoerythrin (PE), and CD80-FITC. **a** Comparison of surface marker expression on freshly isolated M0- versus M1- versus M2-differentiated macrophages from HD and patients with RA presented as the percentage of positively stained cell populations. **b** Quality of surface marker expression in M1- versus M2-differentiated macrophages from HD and patients with RA was analyzed by mean fluorescence intensity (MFI) and presented as a box plot (*upper panel*) and with representative histograms (*lower panel*; *light gray area* = unstained cells, *dark full line* = M1, *dotted line* = M2). MFI was calculated as ΔMFI = MFI_specific surface marker_ − MFI_corresponding unstained control_ and normalized to the basal MFI of unstained control cells. **c** Effect of TLR or interferon (IFN)-γ/lipopolysaccharide (LPS) stimulation on surface marker expression in M1- or M2-differentiated macrophages from HD compared with patients with RA presented as percentage of positively stained cell populations. *n* = 6, * *p* < 0.05

We first assessed surface maker expression on freshly isolated monocytes and monocyte-derived M1 and M2 macrophages from peripheral blood of HD and patients with RA. To generate human M1-like and M2-like macrophages in vitro, we applied a well-established method by differentiating macrophages from peripheral monocytes in the presence of GM-CSF or M-CSF, respectively [23, 25, 29–32]. The phenotypical analysis confirmed that the generated M1 and M2 populations express distinct subset-specific surface marker profiles. These patterns were comparable in the cells derived from blood of HD or patients with RA (Fig. 1a, *left* versus *right panel*, and Additional file 1) Higher CD14 levels and the expression of CD163 have been reported to represent anti-inflammatory M2 macrophages [20]. In our experimental setup, M-CSF-differentiated M2 macrophages from both conditions displayed positive CD14 and CD163 expression, whereas GM-CSF-differentiated M1 macrophages exhibited significantly lower CD14 levels and expressed hardly any CD163. CD206 was previously described as a marker of differentiated M2 macrophages [33], but, in line with other studies [34, 35], it is also expressed on GM-CSF-polarized M1 macrophages. In contrast, freshly isolated monocytes exhibited only low CD206 expression, though M0 from patients with RA showed higher levels than that from HD. CD86 and CD80 were reported to be present on cytokine-activated M1 [21]. However, in accordance with another study [29], the GM-CSF/M-CSF differentiation method resulted in M2 macrophages that expressed CD86 to a similar degree as M1 or M0, whereas CD80 expression was barely detected in the three tested cell types (data not shown).

A more detailed investigation of individual surface marker expression on M1 and M2 by quantification of signal intensity using MFI measurements (Fig. 1b, *upper* and *lower panels*) also demonstrated comparable profiles in cells derived from HD or patients with RA (Fig. 1b, *left* versus *right panels*). This analysis substantiated that GM-CSF-differentiated M1 macrophages expressed significantly lower levels of CD14 than M2, as described elsewhere [36]. In addition, the MFI values also revealed not only that CD206 was expressed on both polarized subsets (M1 and M2) but also that its expression levels were initially higher in M1 than in M2 macrophages, whereas CD86 showed higher expression on M1 than on M2 (Fig. 1b, *upper* and *lower panels*).

Next, we wanted to study the phenotypical profile of M1 and M2 macrophages in the context of inflammatory conditions such as RA. We therefore analyzed the surface marker expression upon TLR stimulation or treatment with IFN-γ/LPS for 24 h. Importantly, the stimulation of M1 and M2 subsets with TLR ligands for 24 h did not significantly alter the proportion of positively stained cells for all surface markers tested (Fig. 1c). Again, there were no significant differences in the distinct M1 and M2 subsets derived from HD compared with patients with RA regarding the surface marker profiles upon TLR treatment (Fig. 1c). Thus, TLR2 stimulation by Pam3 did not significantly change the classical surface marker profile of M2 macrophages. In addition, the MFI representing the quality and amount of CD14- and CD163-positive staining was not significantly affected by TLR2 engagement in M2 macrophages from HD and patients with RA, thereby confirming the sustained integrity of the anti-inflammatory M2 cell surface phenotype (Additional file 2). In contrast, TLR4 ligand LPS or activation by IFN-γ/LPS showed a trend toward lower MFI of M2-polarized markers CD14, CD163, and CD206 while increasing the signal for M1-specific marker CD86, thus underlining the role of CD86 as a marker of “activated” M1 macrophages (Additional file 2).

### Change of characteristic anti-inflammatory M2 gene markers *HMOX1*, *FOLR2*, and *SLC40A1* following TLR ligand exposure and activation

**Fig. 2:**
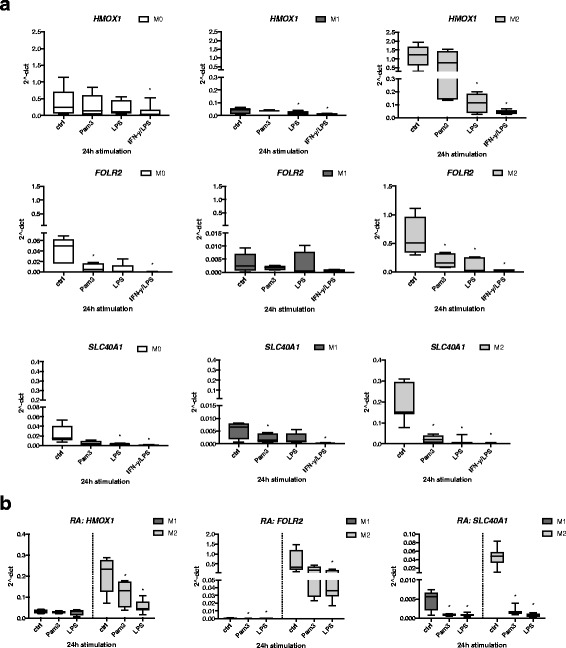
Change of characteristic anti-inflammatory M2 gene markers following Toll-like receptor ligand exposure and activation. **a** M0 (monocytes), M1 (GM-CSF-differentiated), and M2 (M-CSF-differentiated) macrophages were stimulated for 24 h with 300 ng/ml Pam3, 100 ng/ml lipopolysaccharide (LPS), or a combination of interferon (IFN)-γ/LPS (20 ng/ml and 100 ng/ml). Change in gene expression of M2 markers *HMOX1*, *FOLR2*, and *SLC40A1* following stimulation was measured by qRT-PCR. **b** Changes in *HMOX1*, *FOLR2*, and *SLC40A1* gene expression following Pam3 or LPS exposure for 24 h was also measured in M1- and M2-differentiated macrophages derived from blood of patients with rheumatoid arthritis (RA). Values were normalized to *UBC* or *TBP* messenger RNA levels and expressed as 2^−ΔCT^ ± SD. *n* = 4–5, * *p* < 0.05

Besides cell type-specific expression of surface markers, the expression of certain genes also has been shown to characterize anti-inflammatory M2 macrophages [22, 23]. In our experimental system, we confirmed upregulated gene expression of *HMOX1*, *FOLR2*, and *SLC40A1* in M2 compared with M1 derived from both HD and patients with RA (Additional file 3). Next, we investigated whether TLR stimulation had an effect on the expression profile of these gene markers in M1 and M2 macrophages (Fig. 2a). We observed that activation of TLR2 with Pam3 led to a significant downregulation of *FOLR2* and *SLC40A1* gene expression in M2 macrophages, whereas expression of *HMOX1* was only mildly reduced in M2 (Fig. 2a, *right panel*). Remarkably, TLR4 stimulation with LPS or activation with IFN-γ/LPS also significantly downregulated *HMOX1*, *FOLR2*, and *SLC40A1* gene expression after 24 h. Finally, TLR activation or cytokine stimulation in M0 or M1 (Fig. 2a, *left* and *middle panels*) showed an expression pattern of the tested genes similar to that in M2, though their basal expression levels were low or at the limit of detection (Additional file 3). We also tested the observed suppression of these marker genes in M1 and M2 macrophage subsets derived from peripheral blood monocytes of patients with RA. The results showed a comparable gene expression profile and downregulation of *HMOX1*, *FOLR2*, and *SLC40A1* by TLR2 and TLR4 ligands as the HD cells (Fig. 2b).

In summary, these results indicate that TLR2, and to a higher degree TLR4, ligands are able to change the anti-inflammatory M2 gene markers toward an M1-specific expression phenotype.

### Changes in cytokine secretion profile of M0, M1-, and M2-polarized macrophages following TLR ligand exposure and activation

**Fig. 3:**
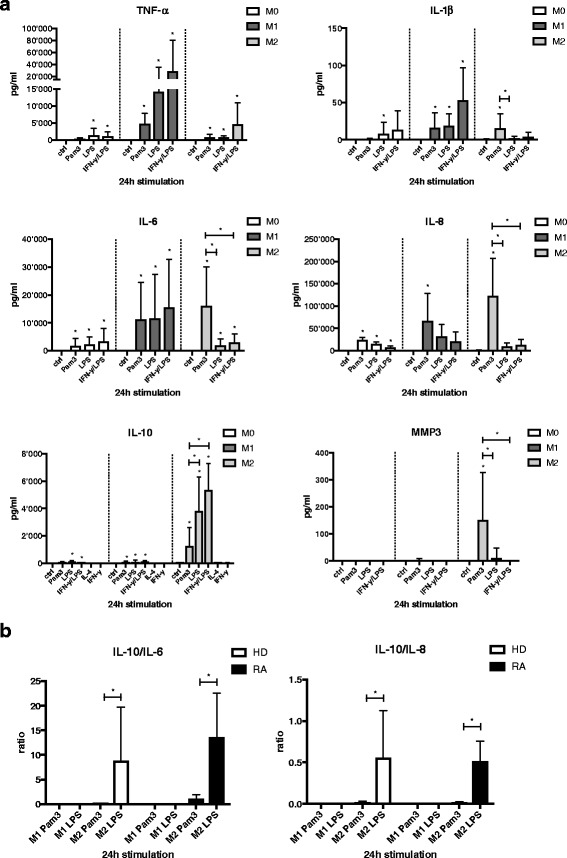
Cytokine profile of M0, M1-, and M2-polarized macrophages following Toll-like receptor ligand exposure and activation. **a** M0 (monocytes), M1 (GM-CSF-differentiated), and M2 (M-CSF-differentiated) macrophages were stimulated for 24 h with 300 ng/ml Pam3, 100 ng/ml lipopolysaccharide (LPS), or a combination of interferon (IFN)-γ/lipopolysaccharide (LPS) (20 ng/ml and 100 ng/ml). Cytokine and matrix metalloproteinase 3 (MMP3) release was measured by enzyme-linked immunosorbent assay, and values are expressed as mean ± SD. *n* = 4–10, * *p* < 0.05. **b** Anti-inflammatory activity was calculated by the ratio of secreted interleukin (IL)-10 to IL-6 or IL-8 and compared in M1- versus M2-differentiated macrophages derived from blood of healthy donors (HD; *open bars*) or patients with rheumatoid arthritis (RA; *black bars*) upon stimulation with 300 ng/ml Pam3 or 100 ng/ml LPS. Values are expressed as mean ± SD. *n* = 7, * *p* < 0.05. *TNF-α* Tumor necrosis factor-α

Previous studies have shown preferential secretion of proinflammatory cytokines by M1 (TNF-α, IL-1β, IL-6, IL-8) and secretion of anti-inflammatory cytokines (e.g., IL-10) by M2 macrophages upon activation [21]. Our experiments confirmed an increased secretion level of the proinflammatory cytokine TNF-α by M1 compared with M0 and M2 following stimulation with TLR ligands or activation with IFN-γ/LPS (Fig. 3a). As expected, M1 macrophages stimulated with TLR ligands Pam3, LPS, or IFN-γ/LPS secreted IL-1β, IL-6, and IL-8 but hardly any anti-inflammatory IL-10. In contrast, IL-10 was abundantly produced by M2 in response to TLR ligands and IFN-γ/LPS, thereby confirming the anti-inflammatory response of M2 macrophages upon activation. Unexpectedly, stimulation of M2 macrophages with Pam3, but not with LPS, strongly induced the production of proinflammatory cytokines IL-1β, IL-6, and IL-8. As a consequence, the ratio of IL-10 to IL-6 or IL-8, an indicator of an anti-inflammatory cytokine profile, was significantly decreased in M2 macrophages by Pam3 stimulation as compared with LPS stimulation (Fig. 3b, *open bars*). The same result was obtained with M1 and M2 macrophages derived from blood of patients with RA (Fig. 3b, *black bars*). Because proteolytic pathways have a critical function in RA development, we were interested in MMP3 secretion upon TLR ligand stimulation in M1 and M2 macrophages derived from blood of HD or patients with RA. We found that TLR2 engagement, but not TLR4, induced a significant MMP3 release only in M2 and not in M0 or M1 (Fig. 3a, Additional file 4).

Therefore, our results indicate that TLR2 engagement in M2 macrophages led to impaired anti-inflammatory activity based on the secretion profile of anti- versus proinflammatory cytokines and might be a source of elevated MMP3 levels found in the pannus and synovium of patients with RA [37].

### *TLR2* and *TLR4* gene expression in M0, M1-, and M2-polarized macrophages following TLR ligand exposure

**Fig. 4:**
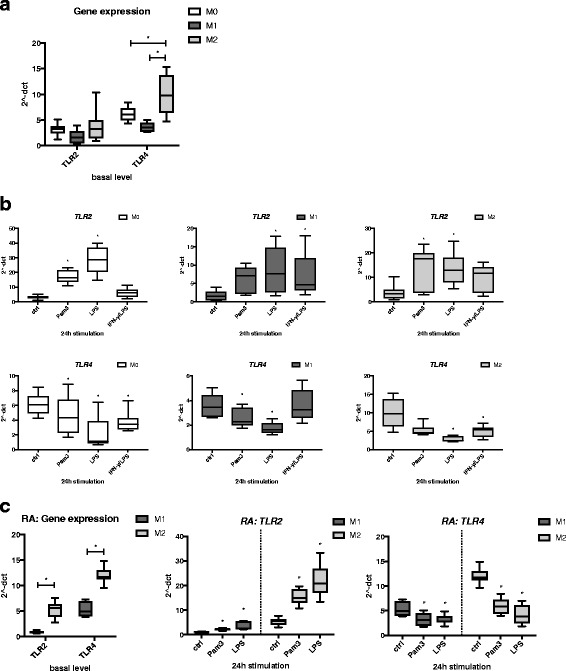
*TLR2* and *TLR4* gene expression in M0, M1-, and M2-polarized macrophages following Toll-like receptor (TLR) ligand exposure. **a** Basal gene expression levels of *TLR2* and *TLR4* were measured in M0 (monocytes), M1 (GM-CSF-differentiated), and M2 (M-CSF-differentiated) macrophages by qRT-PCR. **b** Cells were stimulated for 24 h with 300 ng/ml Pam3, 100 ng/ml lipopolysaccharide (LPS), or a combination of interferon (IFN)-γ/LPS (20 ng/ml and 100 ng/ml), and gene expression of *TLR2* and *TLR4* was measured by qRT-PCR. **c** Basal expression (*left panel*) and changes in *TLR2* and *TLR4* gene expression (*right* and *middle panels*) following Pam3 or LPS exposure for 24 h were also measured in M1- and M2-differentiated macrophages derived from blood of patients with rheumatoid arthritis (RA). Values were normalized to *TBP* and expressed as 2^−ΔCT^ values ± SD. *n* = 4–5, * *p* < 0.05 compared with untreated cells (ctrl)

Next, we investigated the changes in *TLR2* and *TLR4* gene expression in M0, M1, and M2 upon stimulation with TLR ligands. The basal gene expression of *TLR2* did not significantly differ between the cell types, whereas *TLR4* was expressed at a higher level in M2 than in M1 or M0 (Fig. 4a). Interestingly, *TLR2* was upregulated by Pam3 and LPS in M0, M1, and M2. By contrast, *TLR4* was downregulated by LPS in all three cell types (Fig. 4b). As with the previous measurements regarding surface and gene markers or cytokine profiles, we basically found the same tendencies for *TLR2* and *TLR4* expression in macrophages generated from monocytes of patients with RA as those in cells of HD and treated with TLR ligands (Fig. 4c). Interestingly, and in contrast to HD macrophages, the basal gene expression level of TLR2 in RA-derived M2 macrophages was significantly higher than in M1. Taken together, stimulation of M1 and M2 macrophages with TLR ligands Pam3 and LPS resulted in upregulation of *TLR2* gene expression but downregulation of *TLR4*.

### NF-κB, MAPK, and IRF3 activation in M1- and M2-polarized macrophages following TLR ligand exposure

To investigate the underlying cause of the different cytokine secretion profiles in M2 versus M1, we assessed the ability of two different TLR ligands, Pam3 and LPS, to activate NF-κB, IRF3/7, and MAPK pathways in the two macrophage subsets. We performed Western blotting experiments and compared nuclear shuttling of p65 and IRF3/7 as well as phosphorylation of MAPKs p38, ERK1/2, and JNK following stimulation for 30 minutes or 1 h. In concordance with a previous study [38], stimulation of TLR3 by poly(I:C) did not alter NF-κB or MAPK activation in macrophages. We also found that poly(I:C) stimulation showed no significant effect on the tested surface marker expression and cytokine production (data not shown). We therefore used TLR3 engagement by poly(I:C) as ligand control.

**Fig. 5:**
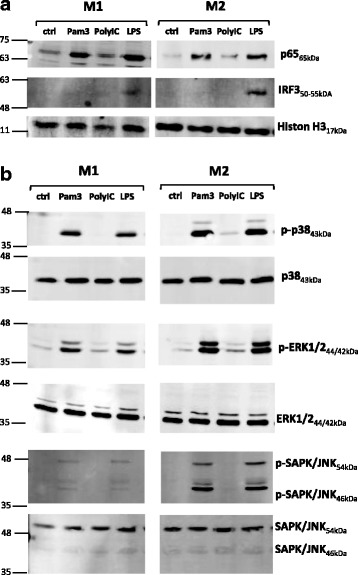
NF-κB, mitogen-activated protein kinase, and interferon regulatory factor 3 activation in M1- and M2-polarized macrophages following Toll-like receptor ligand exposure. **a** M1 (granulocyte-macrophage colony-stimulating factor [GM-CSF]) and M2 (macrophage colony-stimulating factor [M-CSF]) macrophages were stimulated for 1 h with 300 ng/ml Pam3, 10 μg/ml polyinosinic-polycytidylic acid [poly(I:C)], and 100 ng/ml lipopolysaccharide. Nuclear translocation of p65 (NF-κB) and IRF3 was detected by Western blotting. *n* = 3. **b** M1 and M2 macrophages were stimulated for 30 minutes with 300 ng/ml Pam3, 10 μg/ml poly(I:C), and 100 ng/ml LPS. Specific phosphorylation of MAPKs p38, ERK1/2, and c-Jun N-terminal kinase (JNK) was detected by WB. *n* = 2–3

NF-κB was similarly activated in M1 and M2 by TLR2 and TLR4 ligands (Fig. 5a). Only LPS induced nuclear IRF3 shuttling, which was more prominent in M2 than in M1. Furthermore, IRF7 was not detectable, and we had to conclude that there was no activation of IRF7 by any tested TLR ligand in M1 or in M2 in our experimental setting (data not shown). Phosphorylation of MAPKs p38 and ERK1/2 displayed a slightly stronger signal in M2 than in M1 following stimulation by Pam3 and LPS (Fig. 5b). It is noteworthy that phosphorylation of JNK was much more prominent in M2 than in M1 with a preference for the JNK 46 kDa isoform. However, there was no difference between Pam3 and LPS in the activation of the different MAPKs within the respective cell types. Taken together, there were no differences in NF-κB and MAPK activation levels, possibly explaining the higher proinflammatory cytokine secretion in M2 macrophages following Pam3 versus LPS stimulation. However, there was a prominent difference between M1 and M2 macrophages regarding the activation of IRF3 as well as JNK activation by TLR2 and TLR4 ligands.

## Discussion

Macrophages play an important role in the pathogenesis of RA. Depending on the local microenvironment, they can be polarized toward either proinflammatory M1 or anti-inflammatory M2 macrophages [20, 21]. In the present study, we aimed to investigate the phenotypical and functional plasticity of predifferentiated M1 and M2 macrophage subtypes under conditions associated with RA, such as the presence of abundant TLR agonists. By differentiating monocytes from peripheral blood of HD or patients with RA into M1 and M2 macrophages and exposing them to TLR ligands Pam3 and LPS, we anticipated to elucidate the processes that affect infiltrating monocytes in inflamed synovial tissue. Using this experimental design, we demonstrate that M2-polarized macrophages derived from monocytes of HD or patients with RA display an impaired anti-inflammatory activity profile under TLR2 engagement compared with TLR4 stimulation. Thus, following TLR2 stimulation by its ligand Pam3, the M2 population secreted the proinflammatory cytokines IL-6 and IL-8 at levels comparable to Pam3-stimulated M1-polarized macrophages. Despite this shift toward a proinflammatory M1 function, M2 macrophages continued to express the typical M2 cell surface markers CD14 and CD163. However, gene expression of *HMOX1*, *FOLR2*, and *SLC40A1*, three characteristic markers of an anti-inflammatory M2 phenotype, were reduced toward M1 levels, thus correlating with the promoted proinflammatory cytokine profile seen in M2 following TLR2 stimulation. Somehow, unexpectedly, we found that TLR4 stimulation by LPS also led to prominent downregulation of these M2 genetic markers, even though signaling through TLR4 resulted in strong anti-inflammatory activity as measured by ratio of IL-10 to IL-6 and to IL-8 and as expected for M2 macrophages. Thus, in conditions of abundant TLR2 stimulation, a “chimeric” M2 seems to emerge, displaying an M2-like phenotype defined by surface markers while obtaining M1-like functions as defined by genetic markers and cytokine secretion.

As reported in other publications [39], it is possible that ex vivo monocytes from peripheral blood differ in certain aspects between HD and patients with RA. Indeed, we found that basal expression of the surface marker CD206 differed in freshly isolated monocytes from patients with RA compared with HD. However, we found that the discrete macrophage subsets generated from peripheral monocytes of patients with RA displayed similar if not equal subset-specific phenotypical and functional responses upon TLR2 or TLR4 treatment as compared with HD.

To date, in only a few studies have researchers analyzed macrophage subsets in RA [24, 25]. Ambarus et al. [24] compared different surface markers (CD14, CD163, CD68, CD32, CD64, CD200R, CD80) on macrophages in synovial tissue or in monocyte-derived macrophages from RA versus spondyloarthritis. In line with our study, their data indicate that, in an inflammatory environment, there exist macrophages with a mixed M1/M2 phenotype. In a second study, Soler Palacios et al. [25] undertook phenotypic and transcriptomic characterization of ex vivo isolated CD14^+^ RA SF macrophages and compared them with M1 (GM-CSF) and M2 (M-CSF) macrophages generated in vitro. Their presented data showed that RA SF macrophages exhibit a rather mixed phenotype expressing several M1-like proinflammatory markers but also including M2-like markers. Interestingly, several aspects are compatible with our M2-derived “chimeric” macrophages following TLR2 engagement. Thus, they also demonstrated that *FOLR2* and *SLC40A1* gene expression levels in RA SF macrophages were low and corresponded to the generated M1 (GM-CSF) macrophages. Instead, the expression of *HMOX1*, a third genetic marker for an anti-inflammatory M2 phenotype, was similar in RA SF macrophages compared with the generated M2 subset. In our M2 generated macrophages, stimulation with LPS resulted in significant downregulation of *HMOX1*, whereas Pam3 only mildly reduced *HMOX1* expression. Nevertheless, LPS induced strong anti-inflammatory activity with high IL-10/IL-6 and IL-10/IL-8 ratios. These results suggest that low expression levels of *HMOX1* alone cannot discriminate M1 from M2 macrophages in terms of an anti-inflammatory cytokine secretion profile. In addition, RA SF macrophages in the study of Soler Palacios et al. [25] also exhibited a tendency to express several markers apparent in an M2 cell type, such as increased CD14 and CD163 levels (as measured by MFI) or superior IL-10 expression compared with their generated M1 cell type. Our data therefore indicate that, in contrast to what has been conventionally proposed, surface markers as well as individual gene expression markers do not correlate with proinflammatory or anti-inflammatory cytokine expression in M1 and M2 macrophages under inflammatory conditions, which presumably consist of combined TLR2 and TLR4 stimuli. In this context, it is noteworthy that, under certain circumstances, M-CSF-generated macrophages (M2-like) can exhibit proinflammatory activity, as demonstrated by stimulation with ACPA [40] or TLR ligands in combination with IgG [30]. Likewise, Vogelpoel et al. [30] reported synergistic upregulation of proinflammatory cytokines in M2 macrophages exposed to IgG and TLR ligands, which did not differ between macrophages derived from HD or patients with RA. Furthermore, in addition to pathogen-associated molecular patterns (PAMPs), also damage-associated molecular patterns (DAMPs) such as extra domain A fibronectin, tenascin-C, serum amyloid A, high-mobility group box 1 protein, and gp96 are potent agonists on TLRs. All of these DAMPs have been found at elevated levels in synovia from patients with RA [41], and tissue-resident macrophages are potentially exposed to a mixture of PAMP- and DAMP-related ligands. In the context of DAMPs, it is also noteworthy that TLR2, but not TLR4, signaling induces strong MMP3 secretion, which was found to be a critical factor in the progression of cartilage and bone erosion in advanced RA [37].

In our in vitro study, we could demonstrate that in the presence of abundant TLR2 ligands, M2 macrophages derived from peripheral blood of HD or patients with RA lose their anti-inflammatory activity. IL-10 expression is significantly lower in M2 upon TLR2 engagement than with TLR4 stimulation. IL-10 is a major regulator of immunity to infection [42]; it inhibits the activity of Th1 cells, natural killer cells, and macrophages and limits the production of proinflammatory cytokines and chemokines. ERK1/2 is part of the signaling cascade that is activated in macrophages and promote the production of IL-10 [43, 44]. In our study, we observed that both Pam3 and LPS activate ERK1/2, p38, and JNK to a similar extent, despite the differential effect of the applied ligands on pro- and anti-inflammatory cytokine levels. These observations point to a regimen of regulatory steps that govern the inflammatory and anti-inflammatory responses of M1 and M2 macrophages upon TLR stimulation. Thus, TLR stimulation might generate a broad MAPK signaling that then will subsequently be discriminated at different regulatory checkpoints, such as fine-tuning of downstream target gene expression by a specific set of microRNAs. Of note, all investigated MAPKs were activated to a higher degree in M2 than in M1 after stimulation with TLR2 and TLR4 ligands. These observations might have implications for the use of MAPK inhibitors as anti-inflammatory therapy [45] in RA, because administration of such drugs would reduce the function not only of proinflammatory M1 but also of anti-inflammatory M2 subsets.

TLRs have been shown to be highly expressed in rheumatoid synovial tissue or synovial macrophages from patients with RA [12–16], and the stimulation of these receptors plays a role in the pathogenesis of RA [10, 46, 47]. The importance of TLR signaling for the pathogenesis of RA has been suggested by studies with murine arthritis models. Abdollahi-Roodsaz et al. [48] found that development of streptococcal cell wall-induced arthritis in mice was dependent on TLR2 during the acute phase, and this effect shifted to TLR4 dependency during the chronic phase. They also showed that administration of a TLR4 antagonist suppressed clinical and histologic characteristics of arthritis in a mouse model of collagen-induced arthritis [49]. Pierer et al. [50] revealed a significantly lower incidence of collagen-induced arthritis in TLR4-deficient mice. In a model of zymosan-induced arthritis, it was demonstrated that TLR2-deficient mice showed a decrease in early and late phases of joint inflammation [51]. These studies indicate that both receptors play an important role in the development of arthritis.

TLRs are discussed as therapeutic targets for inflammatory diseases but also for cancer [52, 53]. Several agonists and antagonists are under development and are already in different clinical phases. Therapeutic effects of treatment with anti-TLR2 and anti-TLR4 monoclonal antibodies have been investigated in a mouse study against polymicrobial sepsis [54]. Interestingly, a single administration of either anti-TLR2 or anti-TLR4 increased the survival rate and decreased peritoneal, serum, and lung TNF-α levels more efficiently than a combinatorial approach. Our results indicate that therapeutic administration of TLR4 antagonists in RA may result in less beneficial treatment outcomes because the anti-inflammatory activity of M2 macrophages might be impaired by the blockage of TLR4. In addition, depletion of TLR4 signaling would possibly render macrophages more prone to activation of TLR2 by binding of DAMPs present in the synovium of patients with RA. By contrast, the use of TLR2 antagonists as potential RA therapeutics might decrease the release of proinflammatory cytokines from M1-like macrophages while increasing the anti-inflammatory properties of M2-like macrophages by allowing TLR4-mediated IL-10 secretion.

## Conclusions

We show that stimulation with TLR2 but not TLR4 ligands drives M2-polarized macrophages to secrete proinflammatory cytokines and impairs their anti-inflammatory activity. Importantly, the generation of this chimeric M1/M2 macrophage subset upon TLR2 stimulation goes along without major changes in the surface marker profile. Thus, the classical M1/M2 paradigm as based on surface marker expression does not apply to macrophage functions under inflammatory conditions such as RA.

## Additional files

Additional file 1: Figure S1A. Characterization of surface markers on M0, M1-, and M2-polarized macrophages derived from peripheral blood of healthy donors (HD) compared with patients with rheumatoid arthritis (RA). For phenotypical analysis, M0 (ex vivo monocytes), M1 (GM-CSF-differentiated), and M2 (M-CSF-differentiated) macrophages derived from peripheral blood of HD or patients with RA were stained for FACS analysis with fluorescently labeled antibodies CD14-APC-Cy7, CD163-FITC, CD206-BV421, and CD86-PE. Comparison of surface marker expression on freshly isolated M0- or M1- and M2-polarized macrophages from HD (*left panels*) versus RA (*right panels*) presented with representative CD14-to-CD86 (*upper panels*) and CD206-to-CD163 (*lower panels*) density plots. *n* = 6. (DOCX 217 kb)

Additional file 2: Figure S1B. Effect of TLR or cytokine stimulation on surface marker expression in M1 versus M2 macrophages derived from peripheral monocytes of healthy donors (HD) and patients with rheumatoid arthritis (RA) based on mean fluorescence intensity (MFI) analysis. M1 (GM-CSF-differentiated) and M2 (M-CSF-differentiated) macrophages were stimulated for 24 h with 300 ng/ml Pam3, 100 ng/ml LPS, or a combination of IFN-γ/LPS. For phenotypical analysis, cells were stained for FACS analysis with fluorescently labeled antibodies CD14-APC-Cy7, CD163-FITC, CD206-BV421, CD86-PE, and CD80-FITC. Individual MFI was calculated as ΔMFI = MFI_specific surface marker_ − MFI_corresponding unstained control_ and normalized to the basal MFI of unstained control cells. *n* = 6, * *p* < 0.05. (DOCX 759 kb)

Additional file 3: Figure S2. Expression of characteristic anti-inflammatory M2 gene markers in M1 and M2 macrophages derived from blood of healthy donors (HD) or patients with rheumatoid arthritis (RA). Basal gene expression of M2 markers *HMOX1*, *FOLR2*, and *SLC40A1* in M0 (monocytes), M1 (GM-CSF-differentiated), and M2 (M-CSF-differentiated) macrophages from HD (*upper panel*) or patients with RA (*lower panel*). Expression was measured by qRT-PCR. Values were normalized to *UBC* mRNA levels and expressed as 2^−ΔCT^ ± SD. *n* = 4–5, * *p* < 0.05. (DOCX 302 kb)

Additional file 4: Figure S3. Cytokine profile of M1- and M2-polarized macrophages derived from blood of patients with rheumatoid arthritis (RA) following TLR ligand exposure and activation. M1 (GM-CSF-differentiated) and M2 (M-CSF-differentiated) macrophages were stimulated for 24 h with 300 ng/ml Pam3 or 100 ng/ml LPS. Cytokine and MMP3 release was measured by ELISA, and values are expressed as mean ± SD. *n* = 5–6, * *p* < 0.05. (DOCX 139 kb)

## Abbreviations

ACPA: Anticitrullinated peptide antibodies
ANOVA: Analysis of variance
CD: Cluster of differentiation
DAMP: Damage-associated molecular pattern
DTT: Dithiothreitol
ELISA: Enzyme-linked immunosorbent assay
ERK: Extracellular signal-regulated kinase
FACS: Fluorescence-activated cell sorting
FITC: Fluorescein isothiocyanate
FOLR2: Folate receptor β
GM-CSF: Granulocyte-macrophage colony-stimulating factor
HD: Healthy donors
HEPES: 4-(2-hydroxyethyl)-1-piperazineethanesulfonic acid
HMOX1: Heme oxygenase 1
IFN: Interferon
IgG: Immunoglobulin G
IL: Interleukin
IRF: Interferon regulatory factor
JNK: c-Jun N-terminal kinase
LPS: Lipopolysaccharide
MAPK: Mitogen-activated protein kinase
M-CSF: Macrophage colony-stimulating factor
MFI: Mean fluorescence intensity
MMP: Matrix metalloproteinase
mRNA: Messenger RNA
PAMP: Pathogen-associated molecular pattern
PMSF: Phenylmethylsulfonyl fluoride
Poly(I:C): polyinosinic-polycytidylic acid
PVDF: Polyvinylidene difluoride
RA: Rheumatoid arthritis
SAPK: Stress-activated protein kinase
SF: Synovial fluid
SLC40A1: Solute carrier family 40 member 1
TBP: TATA-box binding protein
TBS: Tris-buffered saline
TBS-T: Tris-buffered saline with Tween 20
TLR: Toll-like receptor
TNF-α: Tumor necrosis factor-α
UBC: Ubiquitin C
WB: Western blotting
