# Supplementary material for: TLR2 stimulation impairs anti-inflammatory activity of M2-like macrophages, generating a chimeric M1/M2 phenotype
Source: Arthritis Res Ther. 2017 Nov 2;19:245. doi: 10.1186/s13075-017-1447-1 (PMC5667453; doi:10.1186/s13075-017-1447-1)
Supplement: Supplementary file 1 — Characterization of surface markers on M0, M1-, and M2-polarized macrophages derived from peripheral blood of healthy donors (HD) compared with patients with rheumatoid arthritis (RA). For phenotypical analysis, M0 (ex vivo monocytes), M1 (GM-CSF-differentiated), and M2 (M-CSF-differentiated) macrophages derived from peripheral blood of HD or patients with RA were stained for FACS analysis with fluorescently labeled antibodies CD14-APC-Cy7, CD163-FITC, CD206-BV421, and CD86-PE. Comparison of surface marker expression on freshly isolated M0- or M1- and M2-polarized macrophages from HD (left panels) versus RA (right panels) presented with representative CD14-to-CD86 (upper panels) and CD206-to-CD163 (lower panels) density plots. n = 6. (DOCX 217 kb) [file 13075_2017_1447_MOESM1_ESM.docx]

**Additional file 1**

**
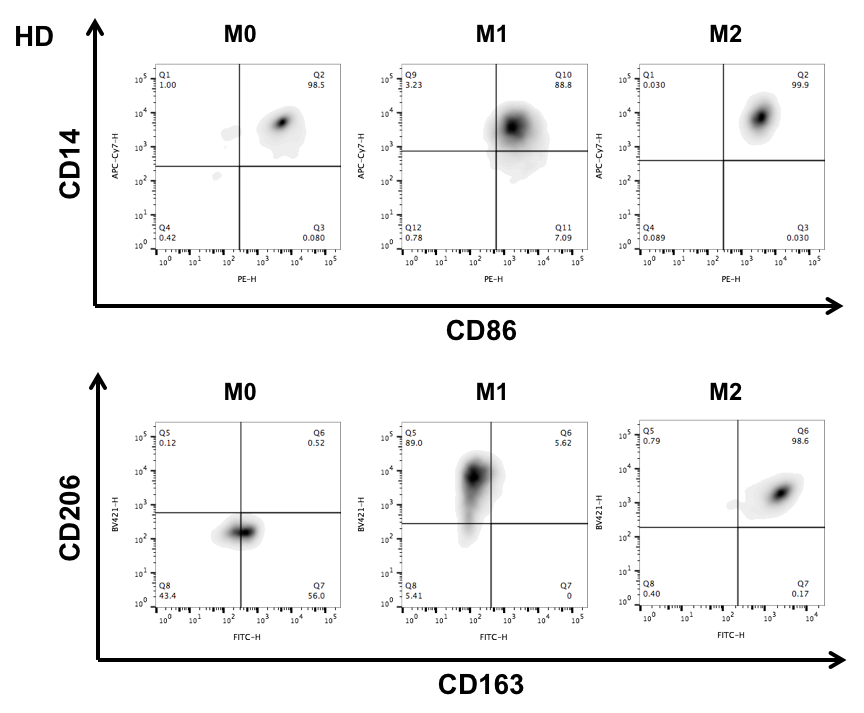

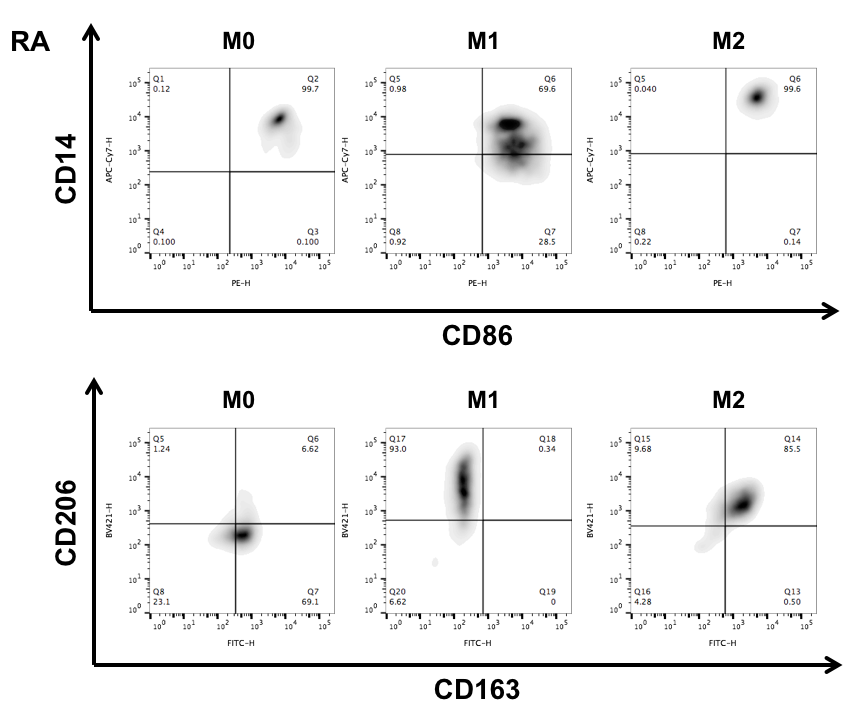
**

**Figure S1A: Characterization of surface markers on M0, M1- and M2-polarized macrophages derived from peripheral blood of healthy donors (HD) compared to rheumatoid arthritis (RA) patients**

For phenotypical analysis, M0 (ex vivo monocytes), M1 (GM-CSF) and M2 (M-CSF) differentiated macrophages derived from peripheral blood of HD or RA patients were stained for FACS analysis with fluorescently labeled antibodies CD14-APC-Cy7, CD163-FITC, CD206-BV421 and CD86-PE. Comparison of surface markers expression on freshly isolated M0 or M1- and M2 polarized macrophages from HD (left panels) vs RA (right panels) presented with representative CD14-to-CD86 (upper panels) and CD206-to-CD163 (lower panels) density plots. N=6.
