# Supplementary material for: TLR2 stimulation impairs anti-inflammatory activity of M2-like macrophages, generating a chimeric M1/M2 phenotype
Source: Arthritis Res Ther. 2017 Nov 2;19:245. doi: 10.1186/s13075-017-1447-1 (PMC5667453; doi:10.1186/s13075-017-1447-1)
Supplement: Supplementary file 2 — Effect of TLR or cytokine stimulation on surface marker expression in M1 versus M2 macrophages derived from peripheral monocytes of healthy donors (HD) and patients with rheumatoid arthritis (RA) based on mean fluorescence intensity (MFI) analysis. M1 (GM-CSF-differentiated) and M2 (M-CSF-differentiated) macrophages were stimulated for 24 h with 300 ng/ml Pam3, 100 ng/ml LPS, or a combination of IFN-γ/LPS. For phenotypical analysis, cells were stained for FACS analysis with fluorescently labeled antibodies CD14-APC-Cy7, CD163-FITC, CD206-BV421, CD86-PE, and CD80-FITC. Individual MFI was calculated as ΔMFI = MFIspecific surface marker − MFIcorresponding unstained control and normalized to the basal MFI of unstained control cells. n = 6, * p < 0.05. (DOCX 759 kb) [file 13075_2017_1447_MOESM2_ESM.docx]

**Additional file 2**

**CD14**

**RA**

**HD**

**CD163**

**CD86**

**CD206**

**CD80**

**Figure S1B: Effect of TLR or cytokine stimulation on surface marker expression in M1- vs M2-macrophages derived from peripheral monocytes of healthy donors (HD) and rheumatoid arthritis (RA) patients based on Mean Fluorescence Intensity (MFI) analysis**

M1 (GM-CSF) and M2 (M-CSF) differentiated macrophages were stimulated for 24 hours with 300 ng/ml Pam3, 100 ng/ml LPS or a combination of IFN-γ/LPS. For phenotypical analysis, cells were stained for FACS with fluorescently labeled antibodies CD14-APC-Cy7, CD163-FITC, CD206-BV421, CD86-PE and CD80-FITC. Individual MFI was calculated as ΔMFI = MFI _specific surface marker_ – MFI _corresponding_ _unstained control_ and normalized to the basal MFI of unstained control cells. N=6, * p<0.05.
