# Supplementary material for: TLR2 stimulation impairs anti-inflammatory activity of M2-like macrophages, generating a chimeric M1/M2 phenotype
Source: Arthritis Res Ther. 2017 Nov 2;19:245. doi: 10.1186/s13075-017-1447-1 (PMC5667453; doi:10.1186/s13075-017-1447-1)
Supplement: Supplementary file 3 — Expression of characteristic anti-inflammatory M2 gene markers in M1 and M2 macrophages derived from blood of healthy donors (HD) or patients with rheumatoid arthritis (RA). Basal gene expression of M2 markers HMOX1, FOLR2, and SLC40A1 in M0 (monocytes), M1 (GM-CSF-differentiated), and M2 (M-CSF-differentiated) macrophages from HD (upper panel) or patients with RA (lower panel). Expression was measured by qRT-PCR. Values were normalized to UBC mRNA levels and expressed as 2−ΔCT ± SD. n = 4–5, * p < 0.05. (DOCX 302 kb) [file 13075_2017_1447_MOESM3_ESM.docx]

**Additional file 3**

**Figure S2: Expression of characteristic anti-inflammatory M2 gene markers in M1 and M2 macrophages derived from blood of healthy donors (HD) or rheumatoid arthritis (RA) patients**

Basal gene expression of M2 markers *HMOX1*, *FOLR2* and *SLC40A1* in M0 (monocytes), M1 (GM-CSF) and M2 (M-CSF) differentiated macrophages from HD (upper panel) or RA (lower panel). Expression was measured by qRT-PCR. Values were normalized to *UBC* mRNA levels and expressed as 2^-ΔCT^ ± S.D. N=4-5, * p<0.05.
