# Supplementary material for: TLR2 stimulation impairs anti-inflammatory activity of M2-like macrophages, generating a chimeric M1/M2 phenotype
Source: Arthritis Res Ther. 2017 Nov 2;19:245. doi: 10.1186/s13075-017-1447-1 (PMC5667453; doi:10.1186/s13075-017-1447-1)
Supplement: Supplementary file 4 — Cytokine profile of M1- and M2-polarized macrophages derived from blood of patients with rheumatoid arthritis (RA) following TLR ligand exposure and activation. M1 (GM-CSF-differentiated) and M2 (M-CSF-differentiated) macrophages were stimulated for 24 h with 300 ng/ml Pam3 or 100 ng/ml LPS. Cytokine and MMP3 release was measured by ELISA, and values are expressed as mean ± SD. n = 5–6, * p < 0.05. (DOCX 139 kb) [file 13075_2017_1447_MOESM4_ESM.docx]

**Additional file 4**

**Figure S3: Cytokine profile of M1- and M2-polarized macrophages derived from blood of rheumatoid arthritis (RA) patients following TLR ligand exposure and activation**

M1 (GM-CSF) and M2 (M-CSF) differentiated macrophages were stimulated for 24 hours with 300 ng/ml Pam3 or 100 ng/ml LPS. Cytokine and MMP3 release was measured by ELISA and values are expressed as mean ± S.D. N=5-6, * p<0.05.
